# Effects of Stormwater and Snowmelt Runoff on ELISA-EQ Concentrations of PCDD/PCDF and Triclosan in an Urban River

**DOI:** 10.1371/journal.pone.0151756

**Published:** 2016-03-17

**Authors:** Magdalena Urbaniak, Adrianna Tygielska, Kinga Krauze, Joanna Mankiewicz-Boczek

**Affiliations:** 1 European Regional Centre for Ecohydrology of the Polish Academy of Sciences, Tylna 3, 90-364, Lodz, Poland; 2 Department of Applied Ecology, Faculty of Biology and Environmental Protection, University of Lodz, Banacha 12/16, 90-237, Lodz, Poland; NERC Centre for Ecology & Hydrology, UNITED KINGDOM

## Abstract

The aim of the study was to determine the effects of stormwater and snowmelt runoff on the ELISA EQ PCDD/PCDF and triclosan concentrations in the small urban Sokołówka River (Central Poland). The obtained results demonstrate the decisive influence of hydrological conditions occurring in the river itself and its catchment on the quoted PCDD/PCDF ELISA EQ concentrations. The lowest PCDD/PCDF values of 87, 60 and 67 ng EQ L^-1^ in stormwater, the river and its reservoirs, respectively, were associated with the highest river flow of 0.02 m^3^ s^-1^ and high precipitation (11.2 mm) occurred five days before sampling. In turn, the highest values of 353, 567 and 343 ng EQ L^-1^ in stormwater, the river and its reservoirs, respectively, were observed during periods of intensive snow melting (stormwater samples) and spring rainfall preceded by a rainless phase (river and reservoir samples) followed by low and moderate river flows of 0.01 and 0.005 m^3^ s^-1^. An analogous situation was observed for triclosan, with higher ELISA EQ concentrations (444 to 499 ng EQ L^-1^) noted during moderate river flow and precipitation, and the lowest (232 to 288 ng EQ L^-1^) observed during high river flow and high precipitation preceded by violent storms. Stormwater was also found to influence PCDD/PCDF EQ concentrations of the river and reservoirs, however only during high and moderate flow, and no such effect was observed for triclosan. The study clearly demonstrates that to mitigate the high peaks of the studied pollutants associated with river hydrology, the increased in-site stormwater infiltration and purification, the development of buffering zones along river course and the systematic maintenance of reservoirs to avoid the accumulation of the studied micropollutants and their subsequent release after heavy rainfall are required.

## Introduction

Urban development has a significant impact on the local environment, as well as the climate and hydro-meteorological processes occurring in the city [1]. Changes in the balance of radiation and heat exchange, emission of pollutants to the air, and the conversion of land have a significant impact on the amount and intensity of rainfall occurring in urban areas. As a result of these phenomena, the total annual precipitation in large agglomerations is generally 5–10% higher than that of the surrounding areas, and for individual storms, the increase in precipitation can be as high as 30% [2]. Although rainwater itself is relatively clean, it becomes contaminated stormwater after washing out pollutants deposited on such surfaces as roofs, streets and sidewalks [3, 4]. This, together with the sealing of the catchment in urban areas, promotes flushing of contaminants previously deposited in the catchment area, and their transport to the lowest-located ecosystems, such as rivers or reservoirs. Snowmelt is another important source of pollutants to the urban watercourse. This is due to the poorer air quality often found in the winter season occurring as a result of coal burning and a lower air temperature, which leads to faster deposition of organic, volatile pollutants on the catchment surface. Moreover, snow accumulates more atmospheric pollutants based on the length of its duration, and hence time of exposure. In consequence, during periods characterized by high rainfall preceded by periods of drought and during the melting of the snow/ice cover, urban rivers become the recipients of a number of pollutants [5–8].

**Fig 1:**
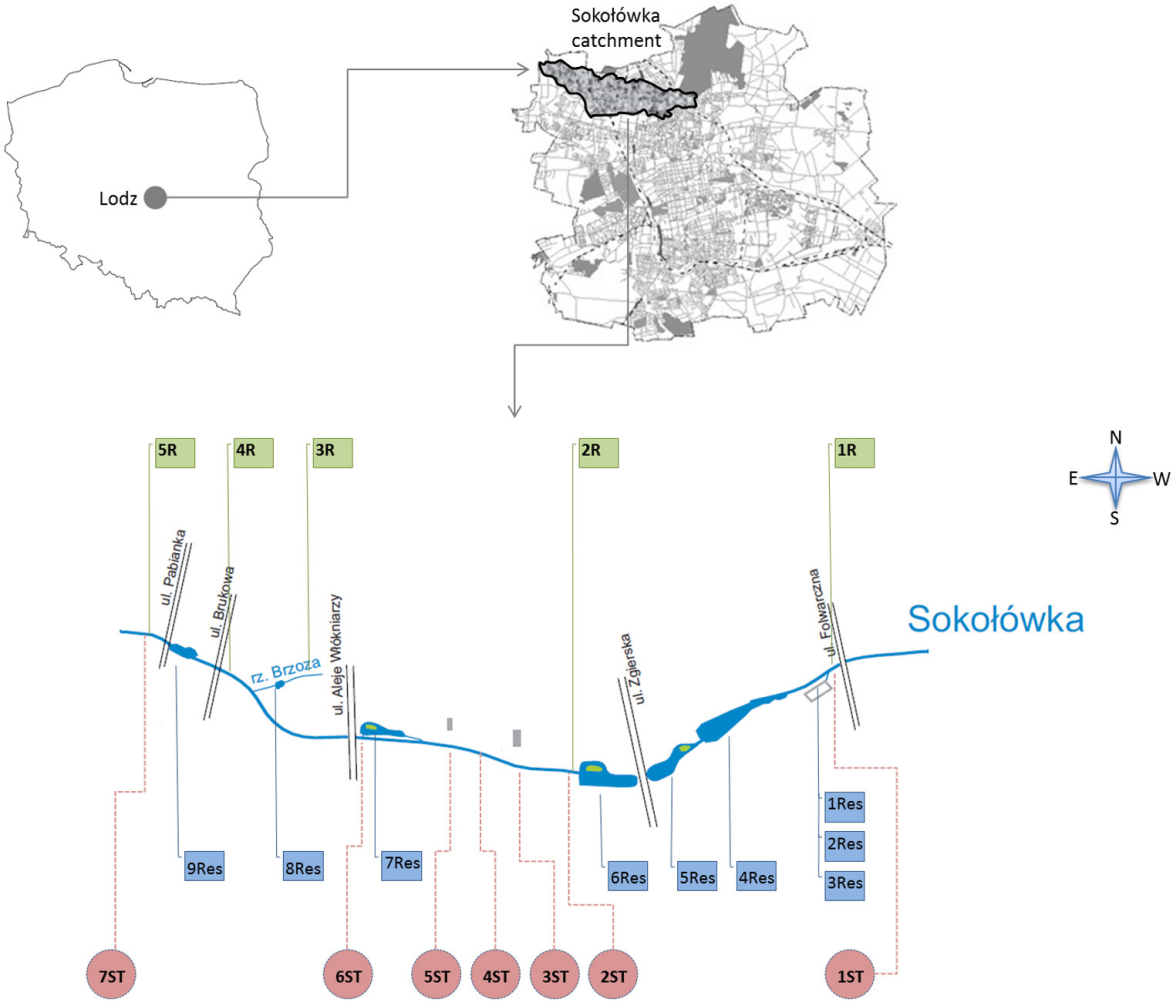
Location of the river, reservoir and stormwater sampling points along the Sokołówka River (1R-5R –river water samples; 1Res-9Res—reservoir water samples; 1ST-7ST—stormwater outflows samples).

Such is the case of the Sokołówka river, a small urban river about 13 km long located in the northwest part of the City of Lodz (Central Poland) in a highly urbanized and industrialized catchment (Fig 1). The main channel was regulated and converted to a collector for stormwater outlets, resulting in the river itself and the reservoirs situated along its continuum acting as receivers for polluted stormwater and illegally discharged wastewater from the surrounding housing estates.

One of the most important groups of pollutants frequently observed in the Sokołowka River is that of organic pollutants, such as polychlorinated dibenzo-*p*-dioxins (PCDDs) and polychlorinated dibenzofurans (PCDFs) [9–15]. These compounds are characterized by a broad spectrum of occurrence in the aquatic environment, as their main source is the input of domestic and industrial sewage, emissions, and atmospheric deposition associated with human activities [16]. Their occurrence in runoff leads to poorer water quality, surface water status and aquatic biodiversity. Due to their high toxicity, persistence and bioaccumulation, PCDDs/PCDFs have been identified as priority hazardous substances which need to be monitored and eliminated from the environment as stated in the Directive of the European Parliament and the Council 2013/39/EC of 12 August 2013 amending Directive 2000/60/EC and 2008/105/EC in respect of priority substances in the field of water policy.

Another substance which may exert a toxic effect on the urban water ecosystem is triclosan (5-chloro-2-(2,4-dichlorophenoxy)phenol): a broad spectrum bactericide used in pharmaceuticals and everyday personal care products [17–19]. Due to its wide usage, triclosan is one of the most frequently-detected pollutants in streams and rivers, being found in 57.6% of US water bodies [20], mostly as an effect of domestic wastewater discharges [19, 21]. As the Sokołówka River is frequently a recipient of untreated wastewater of domestic origin [8, 11, 14, 15] it is of no little importance to determine the amount of triclosan in its waters arising as an effect of such discharges. At the same time, due to the high degree of uncertainty associated with its environmental fate, transport and toxicological effects, triclosan has been classified as an emerging pollutant [22]. Most alarmingly, triclosan, due to its chemical structure, undergoes conversion to highly toxic and persistent 2,8-dibenzodichloro-p-dioxins (2,8-DCDD) [20, 23–25]. This process occurred specially in the aquatic environment exposed to sunlight such as small, shallow rivers and reservoirs [23], wherein between 1 and 12% of 2,8-DCDD is produced as a result of photochemical transformation of triclosan [20, 26]. Consequently, the environmental fate of triclosan as a potential pre-dioxins substance merits special attention and, needs to be monitored, together with PCDDs/PCDFs themselves, especially in small urban rivers which are not only exposed to sunlight but also act as recipients of untreated wastewater [20, 23–29].

The presented study attempts to examine the changes in PCDDs/PCDFs and triclosan concentrations (measured as ELISA-EQ) in the urban river, its reservoirs and the stormwater outlets located along its continuum in relation to the amount of rainfall and river water flow. These findings will allow the effect of stormwater and snowmelt runoff to be determined on PCDD/PCDF and triclosan levels in an urban river.

## Materials and Methods

### Ethics statement

No specific permits were required for the field studies described herein. There was no activity involving endangered or protected species in this study.

### Studied area and sampling

The Sokołówka River (drainage area 45.4 km^2^) is a small river, about 13 km long, located in the northwest part of the City of Lodz (Central Poland) in a highly urbanized and industrialized catchment (Fig 1).

The samples were collected from 21 sampling points located along the river, including five river water sampling points (marked as 1R-5R), nine reservoir sampling points (marked as 1Res-9Res) and seven stormwater outflows (marked as 1ST-7ST) (Fig 1).

The samples were collected during 2013–2014 in five periods of varied meteorological and hydrological conditions:

1. **sampling I**—during the vegetation season, in a period of heavy rainfall occurring five days before sampling, high temperature and low river flow (19^th^ July, 2013);
2. **sampling II**—during the vegetation season, in a period of moderate rainfall occurring five days prior to collection, high temperature and high river flow (3^rd^ September, 2013);
3. **sampling III**—in winter, in a period of rare rainfall occurring in the 5 days prior to collection, very low temperature and moderate river flow (13^th^ December, 2013);
4. **sampling IV**—in winter, in a period of melting snow and ice, low temperature and moderate river flow (12^th^ February, 2014);
5. **sampling V**—in the early spring season, in a period of moderate rain, low temperature and low river flow (15^th^ March, 2014).

In the case of sampling V, it needs to be underlined that sleet fell directly during the sampling procedure itself, which may have an impact on the noted concentrations of pollutants.

The taken samples were filtered with a 0.45 μm pore size Glass Microfiber Filter (GF/C) to remove the suspension, which might negatively affect the concentration of PCDDs/PCDFs and triclosan. After filtration, the samples were stored at -20°C for further analysis.

### Analysis of PCDDs/PCDFs

The assays used in the study were purchased from Abraxis LLC (Warminster, USA). The Abraxis Dioxin/Furan ELISA is an indirect enzyme-linked imunosorbent assay (ELISA) for the screening of PCDDs/PCDFs in water, soil and sediment samples.

Briefly, an aliquot (125 μL) containing one of six calibration standards (0, 2.5, 5, 10, 25 and 50 ng L^-1^), a positive control (3 ng L^-1^) or a sample was mixed with an equal amount (125 μL) of antibody solution and incubated in a glass tube for 60 minutes. After incubation, an aliquot (100 μL) from each vial was transferred to an antigen-coated well in a 96-microwell plate, and incubated for 60 minutes. The content of each well was decanted to remove the solution containing any unbound reagents. Each well was washed four times using 1x washing buffer solution. In the next step, an aliquot (100 μL) of enzyme conjugate solution was added to each well and incubated for 30 minutes. After incubation, the contents of the wells were decanted, and each well was washed four times using 1x washing buffer solution. Following the wash step, an aliquot (100 μL) of chromogenic enzyme substrate solution was added to each well and the plate was incubated for 20 minutes in the dark. In the final step, an aliquot (100 μL) of stop solution was added into each well. The absorbance was measured at 450 nm using a Labsystems Multiskan RC 351 spectrophotometer. The concentrations of the samples were determined using a standard curve and presented as ELISA-equivalency (ELISA-EQ) values.

It needs to be emphasized that the antibody used in ELISA is not only capable of binding to the majority of toxic congeners of PCDDs/PCDFs but also has cross-reactivity with other less toxic PCDD/PCDF congeners. Therefore, ELISA has the potential to be used as an indicator of the total PCDD/PCDF toxicity (named as ELISA-EQ) of the given sample. However, it is not suitable for selective detection and quantification of individual congeners, as this is performed using GC-HRMS. Many studies worldwide have compared the performance of ELISA and GC-HRMS in this regard [30–32], and while the term ELISA-EQ has been suggested to refer to the potency of a sample, GC-HRMS is used to identify the actual toxicity of a sample. Nevertheless, it is possible to convert the GC-HRMS values into ELISA-EQ concentrations using ELISA cross-reactivity factors [30]. Sugawara et al. [31] report the correlation coefficient between ELISA and GC-HRMS analysis to be 0.91, while Nichkova et al. [32] report a correlation coefficient of 0.97. In addition, Van Emon et al. [33] obtained a high concordance between the ELISA and GC/MS methods among environmental samples.

### Analysis of triclosan

The concentration of triclosan was analyzed using a commercial Abraxis Triclosan Assay kit purchased from Abraxis LLC (Warminster, USA). The Abraxis Triclosan Assay applies the principles of ELISA to the determination of triclosan and triclosan methyl. According to Kantiani et al. [34], magnetic particle-based immunoassay can be considered a sensitive and accurate screening tool, suitable for application in water and wastewater analysis, as long as the regression coefficient between this technique and chromatographic analysis was R^2^ = 0.96.

An aliquot (250 μL) of one of four calibration standards (0, 25, 100, 1000 ng L^-1^), positive control (50 ng L^-1^) or a sample was mixed with an aliquot (500 μL) of the triclosan antibody-coupled paramagnetic particles and incubated for 30 minutes. After incubation, an aliquot (250 μL) of triclosan enzyme conjugate was added into each tube and again incubated for 30 minutes. At the end of the incubation period, a magnetic field was applied to hold the paramagnetic particles in the tube and allow the unbound reagents to be decanted. After separation, each tube was washed two times using 1mL of washing solution. Then, the separation rack was removed, an aliquot (500 μL) of color solution was added to each tube and incubated for 20 minutes. Finally, an aliquot (500 μL) of stopping solution was added. The absorbance was measured at 450 nm using a spectral differential imaging (SDI) photometer, and the concentrations of the samples were determined using a standard curve.

### Quality control

Each analytical batch contained a sample blank, a control sample of known concentration (3 ng EQ L-1in the case of PCDDs/PCDFs and 5 ng EQ L^-1^ in the case of triclosan), calibration standards and samples. The precision was verified by duplicate analyses and the test reproducibility was measured using coefficient of variation (CVs). In the case of PCDDs/PCDFs, the CVs should be lower than 12% for calibration standards and lower than 15% for samples; the triclosan CVs should be in turn below 10%. If the CVs exceeded the above values, the whole procedure was repeated in order to achieve good quality of the obtained results. The least delectable dose (LDD), estimated as 90%B/Bo, is 2.5 ng EQ L^-1^ for PCDDs/PCDFs and 20.0 ng EQ L^-1^ for triclosan. Samples showing a concentration lower than LDD were considered to be negative.

### Analysis of hydro-meteorological parameters

#### On line flow monitoring

The used flow module (Isco 2150 Area Velocity Flow Module) uses continuous wave Doppler technology to measure mean velocity. The sensor transmits continuous ultrasonic waves and measures the frequency shift of returned echoes reflected by air bubbles or particles in the flow.

#### Meteorological data

To complement the meteorological database, publicly available values for 24-hour precipitation and air temperature for 2013 and 2014 was used. The data was obtained from weather station no. 124650 (http://www.tutiempo.net/en/Climate/LODZ/124650.htm).

### Statistics

Statistica 8.0 for Windows (Statsoft) was used for all statistical analyses. The Pearson correlation coefficient was used to identify the correlations between the obtained PCDD/PCDF and triclosan ELISA-EQ concentrations and hydrological condition (water river flow and precipitation). Relationships were regarded as significant for p >0.05.

## Results

### Hydrological and meteorological conditions

The hydrological and meteorological conditions observed during the five sampling campaigns vary in terms of temperature, precipitation and river flow.

**Fig 2:**
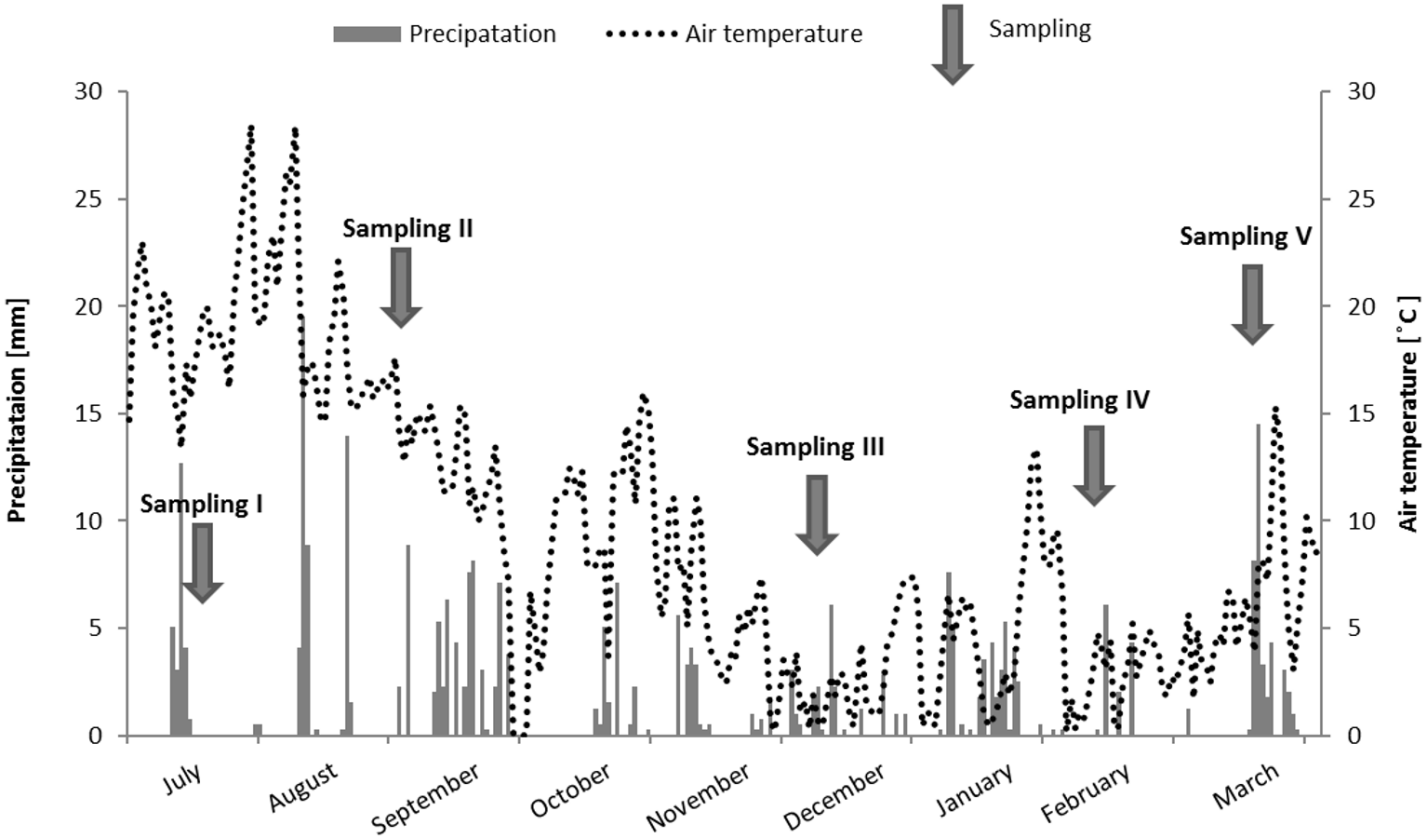
Distribution of precipitation and temperature over the study period.

In terms of air temperature, the highest value of 19.4°C was noted during sampling I (19^th^ July, 2013). It decreased to 12.6°C during sampling II and reached its lowest values during sampling periods III (13^th^ December, 2013) and IV (12^th^ February, 2014): 2.3°C and 2.6°C, respectively. The final sampling period (15^th^ March, 2014) was characterized by higher temperature of 6.5°C (Fig 2).

The monthly precipitation ranged from 12.7 mm in December to 48.5 mm in September. However, the rainfall which occurred five days before the samplings were the heaviest in the case of sampling I (17.52 mm), decreased to 11.2 mm and 8.9 mm during sampling II and III, respectively, and reached the lowest value of 6.4 mm during sampling period IV (however, the intensive snow melting occurred during this time). The last collection was also characterized by low amount of rainfall of 8.4 mm—nevertheless the monthly precipitation reached 39.9 mm, and the samples were collected directly during the rain and snowfall (Fig 2).

**Fig 3:**
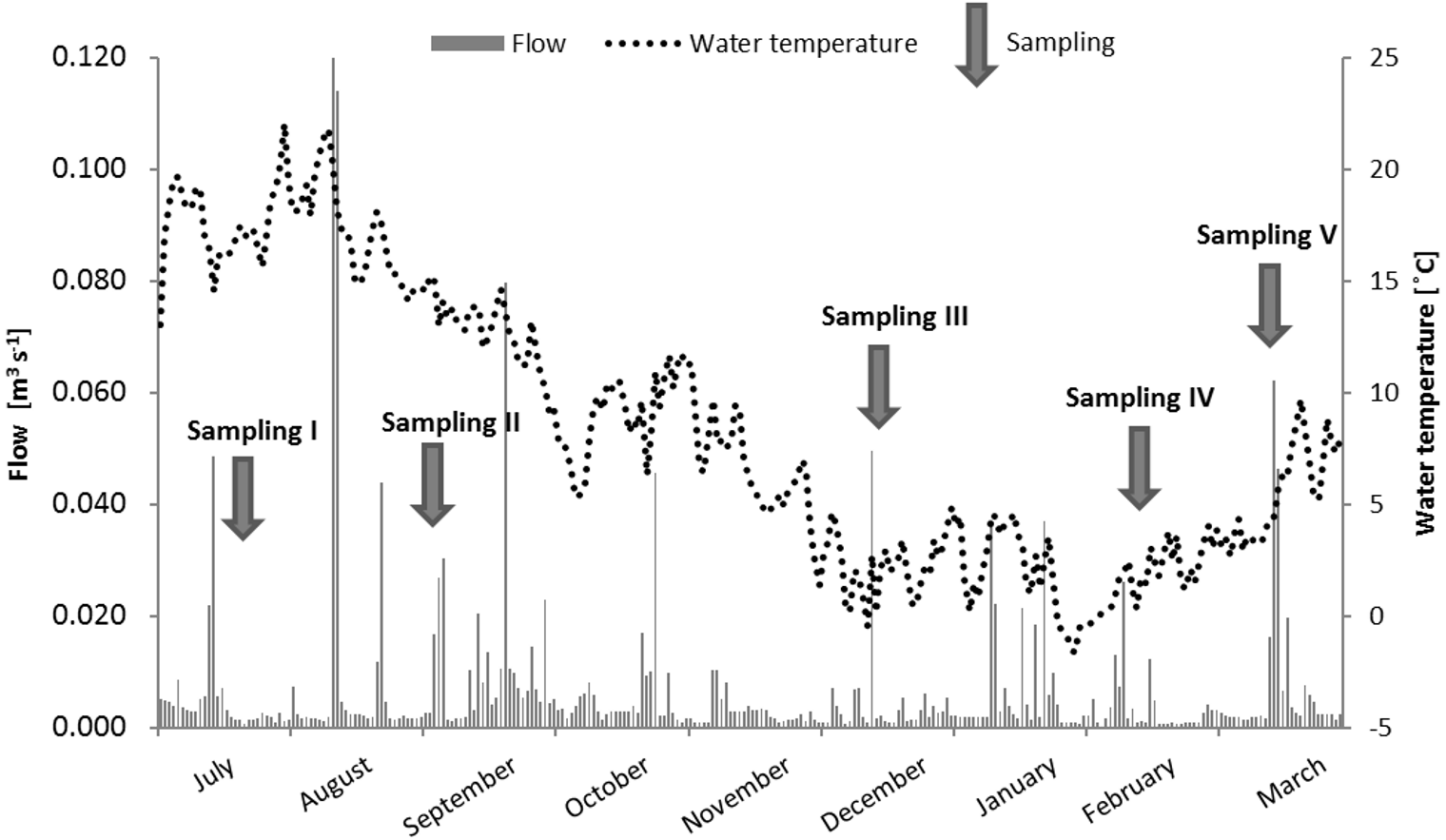
River flow and river water temperature over the study period.

With regard to the average river flow, the lowest values of 0.004 m^3^ s^-1^ occurred in the winter season (sampling III and IV); whereas the highest average monthly flow of 0.011 m^3^ s^-1^ was noted during sampling II (3^rd^ September, 2013) as a result of the high precipitation, which occurred in September (Fig 2). Increased mean monthly river flow of 0.007 m^3^ s^-1^ was also noted during the final sampling (15^th^ March, 2014) while sampling I was characterized by a low value of 0.005 m^3^ s^-1^ (Fig 3), despite the high monthly precipitation of 27 mm (Fig 2).

Despite the average monthly flow, an important task was to calculate the average flow occurring five days prior to each sampling. In this case, the highest flow rates were noted for sampling period II (0.02 m^3^ s^-1^) and the lowest for sampling periods I and V (0.004 and 0.005 m^3^ s^-1^). Moderate flow occurred for sampling periods III and IV (0.01 m^3^ s^-1^) (Fig 3).

### Variation in ELISA-EQ concentrations of PCDDs/PCDFs

**Fig 4:**
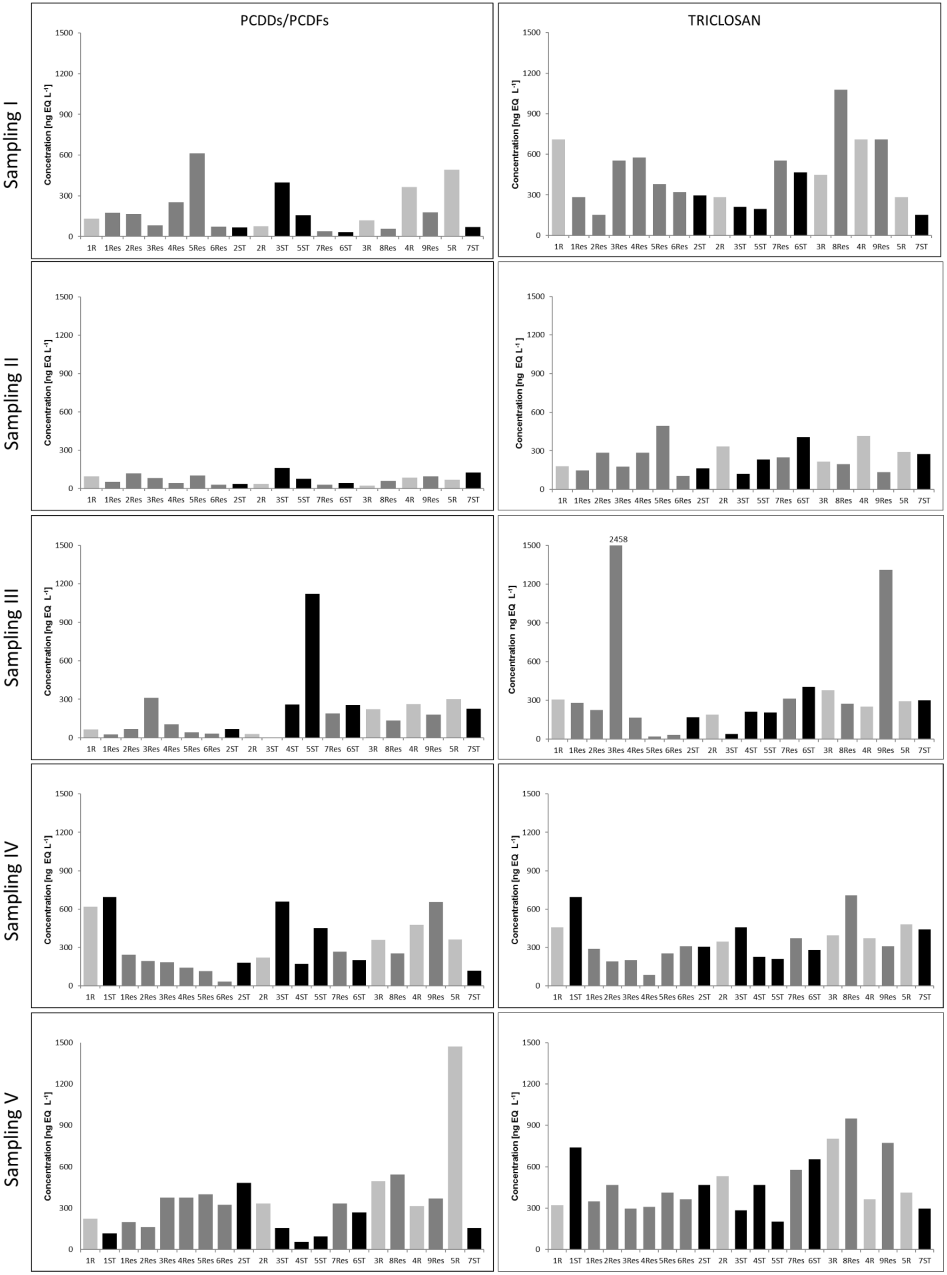
ELISA-EQ concentrations of PCDDs/PCDFs and triclosan noted during the 5 sampling periods.

**Fig 5:**
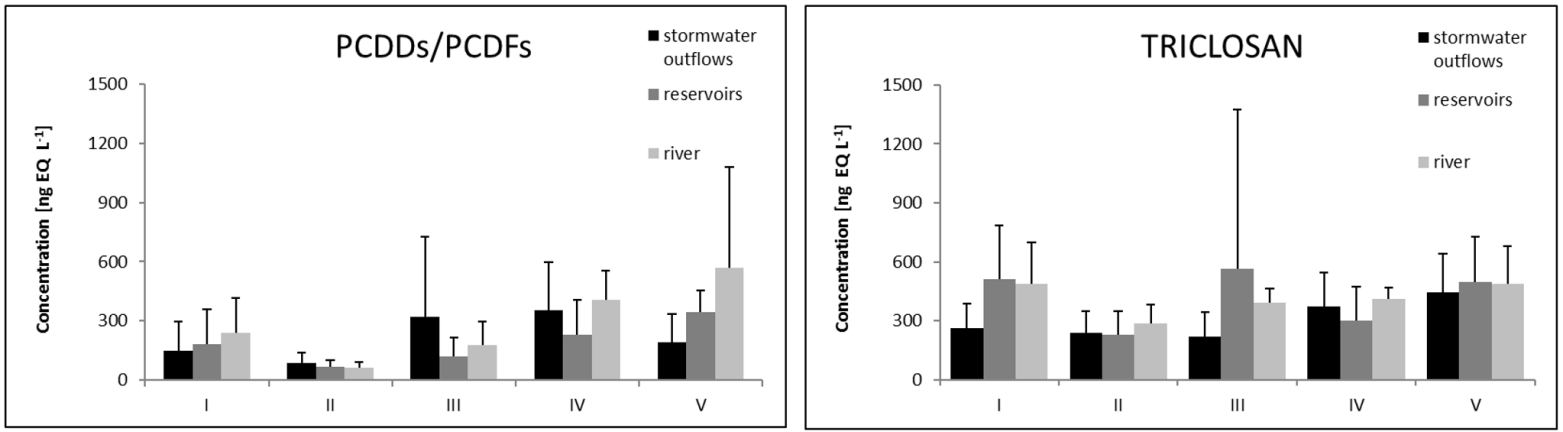
Average ELISA-EQ concentrations of PCDDs/PCDFs and triclosan during the 5 sampling periods.

The results from sampling period I (19^th^ July, 2013), characterized by intense rainfall five days prior to collection and high air temperature were found to vary widely from 34 to 612 ng EQ L^-1^ depending on the sampling point, with the highest concentration observed in the reservoir water sample (5Res) and the lowest in stormwater (6ST- 34 ng EQ L^-1^) (Fig 4). The mean PCDDs/PCDFs concentration during this period was found to be 186 ng EQ L^-1^, while the average concentrations based on sample type were 237 ng EQ L^-1^ for river water samples, 181 ng EQ L^-1^ for reservoir water samples, and only 146 ng EQ L^-1^ for stormwater samples (Fig 5).

In contrast, the results from sampling period II (3^rd^ September, 2013), characterized by moderate air temperatures and rainfall and high flow, showed significantly less variation in PCDD/PCDF concentrations, ranging from 21 to 159 ng EQ L^-1^ (Fig 4) with the lowest mean concentration among the five sample periods (69 ng EQ L^-1^). Also, the average concentrations calculated for river water samples (60 ng EQ L^-1^), for reservoir water (67 ng EQ L^-1^) and for stormwater (87 ng EQ L^-1^) were all low (Fig 5).

In the case of sampling period III (13^th^ December, 2013), characterized by the lowest air temperature and low precipitation, a wide range of concentrations from 2.4 (3ST) to 1120 ng EQ L^-1^ (5ST) were observed (Fig 4) with a noticeable increase in the average concentration (194 ng EQ L^-1^) as compared to the previous campaign. Within the sample type, the highest mean concentration was observed in the case of stormwater (322 ng EQ L^-1^), while river samples were around half this value (176 ng EQ L^-1^) and the lowest values were noted for reservoir samples (120 ng EQ L^-1^) (Fig 5).

The highest concentrations throughout whole study period were quoted for the final two sampling periods: IV (February 2014) and V (March 2014). The conditions for this period were characterized by low temperature, with intense snowmelt (February 2014) and rainfall occurring directly during sample collection (March 2014). The resulting average concentrations for both sampling periods (313 ng EQ L^-1^ and 345 ng EQ L^-1^ for periods IV and V, respectively) were fivefold greater than the values quoted during sampling period II (September 2013), and between 1.6 and 1.8 times higher than sampling periods I and III, respectively (Fig 5).

In the case of sampling period IV, the range of obtained values varied from 31 (6Res) to 694 ng EQ L^-1^ (3ST) and was similar to these observed during the sampling period I (Fig 4). Within the mean concentration the highest value was observed for river samples (407 ng EQ L^-1^), a slight decrease was noted for stormwater (353 ng EQ L^-1^) and the lowest concentration was demonstrated by reservoir samples (231 ng EQ L^-1^) (Fig 5). In opposite, the concentrations obtained for the final (V) sampling period showed an increase in the average concentration of PCDDs/PCDFs in river and reservoir samples (567 and 343 ng EQ L^-1^, respectively), while their concentration in stormwater fell to 189 ng EQ L^-1^ (Fig 5).

The statistical analysis revealed a negative correlation obtained between the average river and reservoir PCDDs/PCDFs concentrations (Pearson correlation coefficients: -0.67 and -0.72, respectively), which was similar to the relationship between the average PCDDs/PCDFs concentrations calculated for all the samples (Pearson correlation coefficient– 0.69) (Table 1). Smaller correlation values were obtained for precipitation, with the highest negative correlation coefficient being with stormwater, and the lowest with reservoir samples (Table 1).

**Table 1:** Pearson correlation coefficients between precipitation and flow (measured 5 days prior to sample collection) and ELISA-EQ concentration of PCDDs/PCDFs and triclosan in all the samples, stormwater samples, reservoir samples and river samples.

Parameter	**PCDDs/PCDFs**			
	All	Stormwater	Reservoir	River
Precipitation	**-0.50**	**-0.64**	-0.29	-0.41
Flow	**-0.69**	-0.25	**-0.72**	**-0.67**
	**Triclosan**			
Precipitation	0.14	-0.47	0.24	0.23
Flow	**-0.96**	-0.45	**-0.76**	**-0.99**

### Variation in ELISA-EQ concentrations of triclosan

The obtained results of triclosan analysis in the samples from sampling period I showed a very wide range of concentrations between 151 (3Res and 7ST) and 1,077 ng EQ L^-1^ (8Res) (Fig 4) with the average of 440 ng EQ L^-1^. The highest mean value was noted in the reservoir samples (511 ng EQ L^-1^), a similarly high concentration was observed for river water samples (487 ng EQ L^-1^) and the lowest was recorded in the case of stormwater (264 ng EQ L^-1^) (Fig 5).

In contrast, the values noted during collection period II showed much less divergence in triclosan concentrations, ranging from 107 (6Res) to 496 ng EQ L^-1^ (5Res) (Fig 4) with the lowest mean value of 249 ng EQ L^-1^ of all the 5 periods. The mean concentrations were 288 ng EQ L^-1^ calculated for river water samples only, 232 ng EQ L^-1^ for reservoirs only, and 241 ng EQ L^-1^ for stormwater only (Fig 5).

During the sampling period III (December 2013), a small increase in average triclosan concentrations was observed (392 ng EQ L^-1^) (Fig 5) with the largest range of the quoted values varying from 21 to 2,457 ng EQ L^-1^ (Fig 4). The average value calculated for river water samples (392 ng EQ L^-1^) was higher than that observed in sampling period II. Similarly, a decline in the concentration of triclosan in the investigated samples of stormwater was observed (222 ng EQ L^-1^) (Fig 5).

In the case of sampling period IV, the range of the obtained results was narrower compared to that of the previous collection period, ranging from 86 to 707 ng EQ L^-1^(Fig 4) with the mean concentration amounted to 351 ng EQ L^-1^. The value calculated only for river water was 410 ng EQ L^-1^, a small decrease was observed for stormwater (373 ng L^-1^), and the lowest value was noted for reservoir samples (302 ng EQ L^-1^) (Fig 5).

The highest concentrations throughout the study period were observed during the final (V) sampling period (March 2014). The meteorological conditions for this period were characterized by a higher mean monthly temperature compared to the two previous sampling periods, and precipitation occurred during the sampling day. The mean concentration of the triclosan calculated for all samples was 478 ng EQ L^-1^, while similar individual mean concentrations determined for river water, reservoir water and stormwater: 486, 499 and 444 ng EQ L^-1^, respectively (Fig 5).

The statistical analysis showed a strong negative correlation between the average concentration of triclosan and the river flow (-0.96), with the highest Pearson correlation coefficient for river samples (-0.99). The relationship between precipitation and triclosan concentrations was much weaker and not statistically significant (Table 1).

## Discussion

### Variation of ELISA-EQ concentrations of PCDDs/PCDFs and triclosan in relation to the amount of rainfall and water flow in the river

The problem of the contamination of Sokołówka River waters by organic compounds such as PCDDs/PCDFs has previously been described by Urbaniak et al. [13], who report that the fate of PCDDs/PCDFs is determined by the hydrological dynamics of the river, which affect the outwashing and downstream transport of allochtonous matter and its associated PCDDs/PCDFs. However, the study was based solely on two sampling periods in winter and summer 2008, and did not reflect the concentrations of the PCDDs/PCDF in stormwater outlets. A more detailed analysis of these compounds in river, reservoir and stormwater under different hydrological conditions was therefore required. The current study was further supplemented by the analysis of triclosan as a possible source of PCDDs/PCDFs in the urban river; while the use of immunoenzymatic assays enabled timely and cost-effective monitoring of the overall PCDD/PCDF and triclosan toxicity of the water samples, and the assessment of the impact of point source pollution (from stormwater outflows) on water quality in the river.

The current study showed that the lowest average concentration of PCDDs/PCDFs (69 ng EQ L^-1^) were recorded during sampling period II, which despite being characterized by moderate monthly precipitation, was also preceded by violent storms in August (Figs 2, 4 and 5), and the highest noted river water flow (monthly: 0.011 m^3^ s^-1^, 5 days: 0.02 m^3^ s^-1^) (Figs 3, 4 and 5). In this case, the reason of the lowest obtained mean PCDDs/PCDFs concentration could be the catchment washing out, as well as the dilution of PCDDs/PCDFs in a large volume of river water.

In contrast, the highest average concentration was recorded during the last two sampling periods (IV– 313 ng EQ L^-1^, and V- 345 ng EQL^-1^), which were characterized by inflow of meltwater (IV) and rain during sample collection (V). In the case of the conditions observed during sampling period IV, the rapid release of contaminants accumulated in the mass of melting snow and ice and their transport with runoff into river ecosystems can affect the significant deterioration of water parameters in Sokołówka River as it was demonstrated in the study of Szklarek et al. [6]. Kawamura and Kaplan [35], Gregor et al. [36], Herbert et al. [37] and Lei and Wania [38] note that snow acts as a source of organochlorine compounds, which are released during snowmelt and cause significant pollution of the water environment. It is also important to emphasize that during winter, the concentration of PCDDs/PCDFs in the atmosphere increases. According to Lohmann and Jones [39], the concentrations of PCDDs/PCDFs and PCBs in the atmosphere can be between 4 and 8 times higher during the winter than the summer. In addition, the rate of photolysis decreases during winter as a result of low exposure to sunlight and a shorter exposure time [39]. All of those factors lead to an enhanced PCDDs/PCDFs concentration in the urban space, thus promoting its flushing into the river ecosystem.

The PCDD/PCDF concentrations identified in the present study are higher than values obtained in other studies worldwide, when converted into ELISA-EQ values using ELISA cross-reactivity factors [30]. For instance ELISA-EQ PCDD/PCDF concentrations noted in a small urban waterway in Osaka (Japan) ranged from 0.0007 to 0.98 pg EQ L^-1^ [40], while those in an urban runoff in Germany were found to be within the range of 0.76 to 4.10 pg EQ L^-1^ [41]. Although much higher concentrations, up to 29 pg EQ L^-1^, were demonstrated in our earlier study conducted in the area of the Sokołowka River [13], they are still lower than those reported in the present study. The reason for such differences is that ELISA-EQ may contain the responses from a range of 210 PCDD/PCDF congeners, many of which are not measured by standard GC/HRMS, which typically only evaluates the sum of 17 toxic PCDDs/PCDFs. Consequently, the results of GC/HRMS analysis are typically underestimated in comparison to ELISA and do not reflect the biological response with regard to the mixture of PCDDs/PCDFs present in the environmental samples [30].

Triclosan is mostly rinsed off during usage and enters the sewage system together with domestic wastewater. It is therefore one of the most frequently detected chemicals in wastewater from households and in rivers receiving untreated or insufficiently treated wastewater [42–48]. Worldwide studies have demonstrated triclosan concentrations in river water ranging from 3 ng L^-1^ to 120 ng L^-1^ [49], while Braush et al. [50] reveal triclosan concentrations of between <0.1 and 2300 ng L^-1^, with a mean of 48 ng L^-1^, in a collection of 710 surface water samples. The concentrations noted in wastewater treatment plant effluents are, however, much higher and range from 42 ng L^-1^ [47] to 5900 ng L^-1^ [44]. As the Sokołówka River water receives both wastewater and stormwater, it is not surprising that the values for triclosan obtained in the present study lie within the range of 21 to 2,457 ng EQ L^-1^. Neighboring housing estates may be a major contributor of triclosan to the Sokołówka River through the infiltration of domestic sewage from septic tanks and illegal discharge of untreated wastewater as demonstrated previously [10–15].

The lowest mean value of triclosan (249 ng EQ L^-1^), as for PCDDs/PCDFs, was recorded during sampling period II. The obtained results coincided with the presence of intense rainfall in August, which affected catchment flushing, the dilution of pollutants from surface runoff, and domestic sewage infiltration from septic tanks and illegal discharges, as noted by Katz et al. [51]. Another cause may be associated with high river flow (Fig 3). Wilson et al. [48] found hydrological conditions such as flow rate to affect the observed concentrations of triclosan. Kolpin et al. [18] demonstrated increased disinfectant concentrations during low flow periods and rapid decreases at high flow. Similarly, our findings reveal a strong negative correlation between river flow and triclosan concentration (Pearson correlation coefficient -0.96) (Table 1).

In contrast, the highest average triclosan concentration was recorded in July (sampling I) and March (sampling V) due to the occurrence of intensive rains preceded by a dry period, which accelerates pollutant runoff from the catchment.

Moderate concentrations, in turn, were identified during sampling periods III and IV. In the former, the presence of low and stable rainfall, combined with the lowest air and water temperatures, may have contributed to the continuous release of pollutants from leaky septic tanks or intentionally discharged wastewater [10–14]. However, in the latter, the obtained concentrations could be due to the inflow of meltwater into the river [6].

### Impact of stormwater on the quoted ELISA-EQ concentrations of PCDDs/PCDFs and triclosan in the river/reservoir water

Generally speaking, there are three global problems with water: too much, too little or too dirty [52]. All of these problems were found to be present during the study on the Sokołówka catchment: firstly, due to its much lower retention capacity, the time needed to reach the flood peaks in the Sokołówka is about one third of that observed in reference catchments; secondly, the unit discharges are three times higher during the cumulation wave; finally, effective precipitation is twice as high as that observed in reference catchments, and the direct discharge coefficient is 1.5–7.6 times that of the reference catchment [53]. In consequence, these dynamic hydrological conditions determine the runoff of pollutants into the river through stormwater overflows.

Worldwide studies have shown that a large number of organic and inorganic substances may be present in stormwater, which additionally may vary from runoff to runoff and from site to site [5, 8, 41, 54–65]. PCDDs/PCDFs EQ concentration in stormwater dominated in sampling periods II and III. During sampling period II, the PCDDs/PCDFs EQ concentration in stormwater was 1.3 and 1.5 times higher than that observed in reservoir and river water samples, respectively, while for period III, the respective concentrations were 2.7 and 1.8 times higher than in the reservoir and river samples. These findings suggest that stormwater was the possible source of PCDDs/PCDFs in the river (Fig 5). This was confirmed by our earlier study, in which the profile of PCDDs/PCDFs in the sediments of Sokołówka reservoirs were found to be similar to those found in urban stormwater street runoff and stormwater sediments [10, 54]. Furthermore, these two sampling periods were characterized by increased river water flow: for example, the highest flow rate occurring just before sampling period III was associated with higher PCDDs/PCDFs concentration in stormwater (Fig 3). Similar results were shown by Gilbreath and McKee [61] with 4- to 28-fold higher PCDDs/PCDFs concentrations noted during storm flow in comparison to low flow. The statistical analysis confirmed the presence of a negative relationship between precipitation and stormwater PCDDs/PCDFs EQ concentration (-0.64), suggesting that the concentration of PCDDs/PCDFs in stormwater undergoes dilution during precipitation.

On the other hand, the results from the final sampling period (V) demonstrated a strong predominance of PCDDs/PCDFs EQ in the river samples: three times higher than that found in stormwater and 1.6 times higher than reservoir water. This may be due to the input of a condensed phase of stormwater, which contains a higher concentration of pollutants than its diluted phase (the “first flush effect”) [8, 62–65]. Zerihun et al. [8], on the basis of an analysis of storm events in the area of the Sokołówka catchment, demonstrated that the peaks of pollutant concentrations precede the maximum flow in the case of rainfall occurring after a period of drought. Fig 3 indicates that the final sampling period took place during the rising stage of the flow peak, thus resulting in a high concentration of pollutants into the river.

Triclosan EQ concentration was lower in stormwater than river samples during all five sampling periods, and even lower than that of the reservoir samples during periods I, III and V (Fig 5). It is also interesting to note that the average triclosan EQ concentration calculated for all samples was the highest during the lowest river water flow measured five days prior to collection (sampling periods I and V, 0.004 and 0.005 m^3^ s^-1^), while its lowest mean value was observed during sampling period II, with the highest river water flow (0.02 m^3^ s^-1^) (Pearson correlation coefficient -0.96). Nonetheless, no such strong relationship was observed for the triclosan concentrations in stormwater (Pearson correlation coefficient -0.45). These findings suggest that concentration of triclosan in the river undergoes dilution during increased flow, as was the case for PCDDs/PCDFs. On the other hand, precipitation led to increased triclosan EQ concentration in the river and reservoirs, but lower concentrations in stormwater (Table 1). This may reflect the impact of domestic wastewater discharged directly to the river during rainfall events, and dilution of triclosan in stormwater due to its higher volume [18].

## Conclusions

This study determines the concentrations and temporal variation of PCDD/PCDF and triclosan content in a small urban river. The obtained results demonstrate that the lowest average ELISA-EQ concentration of PCDDs/PCDFs and triclosan were observed during sampling characterized by high river water flow occurring as an effect of prolonged rain. The highest concentrations, however, were noted in periods of low river flow and occurrence of rainfall/snowmelt proceeded by a rainless phase which resulted in pollutants deposited on the catchment surface being scoured and carried into the urban river. However, stormwater was only found to influence the PCDD/PCDF EQ concentrations of the river and reservoirs during sampling periods II and III, and no such effect was observed for triclosan.

The high concentrations of the micropollutants observed in the water of the Sokołówka River and its reservoirs, compared to other studies indicate the need for more controlled use of those substances on the catchment scale. Furthermore, the dynamic changes in the concentrations found to be associated with river hydrology requires increased in-site stormwater infiltration and purification, the development of buffering zones along water bodies to decrease of inflow from diffused sources, as well as the systematic maintenance of reservoirs to avoid the accumulation of micropollutants and their subsequent release after heavy rainfalls. The study also indicates that monitoring schemes for urban river restoration projects should also trace the dynamics of PCDDs/PCDFs and triclosan, as indicators of urbanization processes in term of domestic wastewater production and pharmaceuticals and personal care products usage.

## Supporting Information

S1 File (XLSX)
